# The pursuit of genetic gain in agricultural crops through the application of machine-learning to genomic prediction

**DOI:** 10.3389/fgene.2023.1186782

**Published:** 2023-08-02

**Authors:** Darcy Jones, Roberta Fornarelli, Mark Derbyshire, Mark Gibberd, Kathryn Barker, James Hane

**Affiliations:** ^1^ Centre for Crop and Disease Management, Curtin University, Perth, WA, Australia; ^2^ Curtin Institute for Computation, Curtin University, Perth, WA, Australia

**Keywords:** machine-learning, genomic prediction, linear-mixed models, crop improvement, genetic gain

## Abstract

Current practice in agriculture applies genomic prediction to assist crop breeding in the analysis of genetic marker data. Genomic selection methods typically use linear mixed models, but using machine-learning may provide further potential for improved selection accuracy, or may provide additional information. Here we describe SelectML, an automated pipeline for testing and comparing the performance of a range of linear mixed model and machine-learning-based genomic selection methods. We demonstrate the use of SelectML on an *in silico*-generated marker dataset which simulated a randomly-sampled (mixed) and an unevenly-sampled (unbalanced) population, comparing the relative performance of various methods included in SelectML on the two datasets. Although machine-learning based methods performed similarly overall to linear mixed models, they performed worse on the mixed dataset and marginally better on the unbalanced dataset, being more affected than linear mixed models by the imposed sampling bias. SelectML can assist in the training, comparison, and selection of genomic selection models, and is available from https://github.com/darcyabjones/selectml.

## Introduction

Machine learning (ML) is rapidly growing in the breadth of potential applications across the agricultural sector, which may include its integration with: real time perimeter surveillance for pest invasion, monitoring crop yield/health via on-farm and remote sensing, precision agriculture using smart farm equipment, supply chain optimisation and traceability, risk and profit forecasting, and pesticide and fungicide management. An area that appears to be less developed than these mostly hardware-centric technologies—but with equal potential for impact—is the application of ML to genomic prediction (GP). GP is the analysis of genetic marker data to enable genetic gain in crop breeding, which can predict desirable traits based on genetic markers, predict high-performing genetic backgrounds and guide selective breeding in a process called genomic selection (GS) (Desta and Ortiz, 2014). Several stages of crop breeding have potential GP applications, including: decision-support in the crossing of wild and elite lines (Prohens et al., 2017), back-crossing (Varshney and Dubey, 2009), or selfing (Frederickson and Kronstad, 1985); and prediction of genetic gain/loss (Voss-Fels et al., 2019). Marker-assisted selection (MAS) is a well-established method used for introducing traits of interest into a breeding population based on association between genotyped markers and specific phenotypes (Ribaut and Hoisington, 1998). Conceptually GP is an extension of MAS, in which numerous markers of unknown phenotypic influence are used to train statistical models on phenotyped plants, and then predict genetic gain/loss for groups of non-phenotyped plants (Whittaker et al., 2000). This can significantly accelerate crop breeding, as phenotypes can be rapidly predicted directly from seed or seedlings, enabling early screening of high/low-performing seed stocks (Crossa et al., 2016).

Linear mixed models (LMMs) (Meuwissen et al., 2001) are commonly used for GP, can handle dependence between samples (e.g., genetic relatedness or environmental similarity), and allow assertion of prior expectations over data distributions. Trait phenotypes are governed by genetic and environmental components, which influences LMMGP model structure. Sources of environmental variation in models may need to be excluded to obtain accurate predictions of genetic gain, or identify high-performing genotypes. An example of building an initial LMM model, then using the BLUPs of genotypic effects as traits for subsequent prediction, is described below. Environmental sources of variation (e.g., resources, stress) can be measured indirectly as blocking factors (e.g., location, season) and modelled as random intercepts, sometimes with autoregressive covariance structures to account for spatial or temporal variation. Direct measurements (e.g., temperature, humidity) can also be used as direct covariates (fixed effects) in the model. Genetic effects can be further divided into additive (simple linear independent), dominance (non-linearity in heterozygote), and epistatic (interactions between combinations of additive and dominant marker contributions) components. As plants respond to variable environmental conditions, genetic components influencing traits can vary, referred to as the genotype-by-environment (GxE) component. Although LMMs can fit complex models with careful consideration of which parameters and interaction terms are included, fully incorporating epistatic and GxE effects may be impractical or impossible to solve. ML-based GP (MLGP) is an alternative to LMMGP for modelling dominance, epistasis and other interaction terms with less explicit specification of interactions and non-linearities. Although not yet fully realised, there is potential with MLGP models to include all parameters and terms and let the model decide which data is important, although this would be unsuitable for smaller datasets. While there is considerable overlap between methods, they are broadly distinguished by their objectives. Statistical models aim to give an explainable model with a direct relationship to biological knowledge and model coefficients (or BLUPs) are estimates of interest. In contrast, ML models apply heuristic methods to obtain a highly accurate prediction without imposing structure upon the coefficients. As a consequence, ML interpretability may be a secondary concern and is often only useful as a qualitative indication of what the model has learnt. In some cases there may be overlap between our definitions of ML and statistical models (e.g., RR-BLUP and GBLUP), however within this study we have considered comparisons between four statistical linear mixed models (LMMs): Bayesian ridge regression (BRR); BayesA, BayesC, and Bayesian LASSO (BL); with six ML methods: Support Vector Regression; k-nearest neighbours (Knn); random forest; Extra trees; Natural Gradient Boosting (NGB); and eXtreme Gradient Boosting (XGB).

Genetic information in breeding programs can be modelled relatively well using simple regularised models, so complex LMMGP and MLGP models may currently not be inherently suited to improving predictive performance. However increasingly larger sample sizes, improved marker selection (i.e., higher proportions of perfect/causal markers), and improved phenotyping may favour complex models and lead to future improvements. Both MLGP and LMMGP can vary from simple and fast to complex and computationally-intensive. MLGP may be more efficient in terms of memory usage, but may have additional hardware requirements (e.g., GPU). Both have issues as datasets increase in scale, however ML methods such as neural networks (NN) using stochastic sampling (e.g., mini-batching) have an advantage with increasing numbers of samples as only sub-samples have to be stored in memory. Reduced phenotypic variance can improve modelling of environmental factors and covariates, allowing removal of sources of variance and significantly improving LMMGP accuracy (Hu et al., 2023), however this has yet to be properly tested with MLGP. LMMs require specification of data combinations the model should use (e.g., epistatic or GxE relationship matrices), whereas ML models may be run without specifying prior assumptions at the cost of reduced interpretability. However, the use of complex MLGP methods under varying levels of epistasis, and dominance is not yet well investigated.

A particular challenge of GP is that the majority of genetic markers are not associated with phenotypes of interest, while most associated markers are only indirectly associated due to close genomic distance to causal loci (syn. linkage disequilibrium, LD). As recombination occurs over generations, markers used to train a GP model become unlinked with causal loci thus GP models lose accuracy (Wientjes et al., 2013). This reliance on LD severely limits the transferability of GP models across populations or successive generations. In ML applications across many other disciplines, the model is typically trained once and may be re-used many times. However for crop-breeding applications, the training population can be constantly increasing as successfully bred genotypes accumulate new phenotype data and models may be retrained as new data becomes available (e.g., at least annually with successive harvests) or if LD becomes lost over time. This restricts current methods to predicting phenotypes within similar populations as to which they were trained. MLGP is typically applied to single environment studies with de-regressed data or uses phenotypes excluding residual environmental factors from the modelling (Crossa et al., 2016; Capblancq et al., 2020). ML has been used to enhance reusability of genomic data (Lung et al., 2020), but there does not yet appear to be ways to improve model transferability. For plant researchers allied with crop-breeding, training of experimental dataset-specific models has very limited applicability for commercial breeding. Therefore, in addition to the identification of perfect causally-linked markers, there are several areas of GP that can be improved. This study explores the potential application of MLGP as an alternative to LMMGP and the use of automated methods for training new MLGP models, with benchmarking relative to commonly-used LMMGP models.

## Methods

To compare relative performance of MLGP and LMMGP, we generated two distinct simulated datasets: a “mixed” dataset with random crosses from 40 parents, and an “unbalanced” dataset unevenly sampled from 5 bi-parental crosses. Artificial marker data was generated with AlphaSimR version 1.1.2 (Gaynor et al., 2020) to simulate two datasets with contrasting population structures: a “mixed” dataset with random crosses from 40 parents; and an “unbalanced” dataset with 5 bi-parental populations with uneven sampling.

SelectML automatically performed feature selection, feature transformation, and hyperparameter optimisation strategies using a scikit-learn (Pedregosa et al., 2011)-compatible API. SelectML used biallelic markers encoded as 0, 1, and 2 (with the heterozygote as 1) as the genetic input, and can optionally use one-hot encoded blocking factors and continuous covariates. SelectML cannot perform multivariate modelling, but can be applied to MET or (standardised) multitrait problems using one-hot encoding with grouping factors. SelectML supports regression, classification, and ranking/ordinal prediction tasks. For regression tasks, target variables may be Z-transformed to fit a standard normal distribution, or quantile scaled to a standard normal or uniform [0, 1] distribution. For ranking tasks we considered all regression target transformations, a cumulative distribution classifier (Burges et al., 2005), and for gradient boosted trees and neural networks we also considered a pairwise ranking scheme where the model is trained to classify whether each sample should be ranked higher than others as described in RankNet (Burges et al., 2005). Feature selection was applied to markers only, using performed using the GWAS program GEMMA version 0.98.3 (Zhou and Stephens, 2012), a minibatched implementation of MultiSURF (Urbanowicz et al., 2018), and by minor allele frequency. The GEMMA model was run in two stages with a kinship matrix calculated in the first stage, and blocking factors and the first three principal components were provided as covariates to the model. The top k markers were selected from the GWAS by lowest *p*-value, from MultiSURF by the highest feature relevance scores, and from MAF by the highest minor allele frequency (i.e., closest to 0.5). Markers were transformed either using one-hot encoding, the minor allele scaling method used before distance matrix calculation described by Van Raden (VanRaden, 2008), a similar minor allele scaling method using the additive NOIA scheme (Álvarez-Castro and Carlborg, 2007), and the first k principal components of the van Raden scaled markers. We also optionally optimised to include a distance matrix as additional features, which includes the van Raden matrix, and the van Raden scaled Manhattan, and Euclidean distance metrics. We also optionally optimised a set of additional non-linear features using an approximate (Williams and Seeger, 2000) kernel method on Van Raden pre-scaled features to provide Laplacian, polynomial (degree = 2), or radial basis function transformations of markers. Both the distance matrices and non-linear features were each scaled to a standard-normal distribution based on their quantiles, and the k best features from each feature set selected using the ANOVA f-score for regression or classification depending on the target task. The model may drop the markers, distance, or non-linear combinations of features entirely and attempt to use the other sets of features instead (e.g., distance matrices only). Grouping factors if provided may be left as one-hot encoded features, or may take the first k principal components. Covariates are scaled to a zero centred range using a Z-score transformation, a robust scaler (centred on the median and scaled by the interquartile range), or using a quantile transformer. Additionally, first, second, or third degree polynomial combinations of the covariates may be included as additional features.

The markers, marker distance, marker non-linear, group, and covariate features were then combined into a single table. Optionally, non-linear combinations of these combined features may be added as additional features using the same kernel functions as described in the non-linear marker interactions, and scaled using a quantile transformer. Finally, these features were applied to a range of models, including k-nearest neighbours, random forests, extra trees, support vector machines (SVM), penalised linear models (i.e., LASSO, ridge, and ElasticNet) using stochastic gradient descent, LARS and LASSO-LARS linear models, extreme gradient boosted trees using XGBoost (Chen and Guestrin, 2016), and bayesian linear genomic prediction models using BGLR (Pérez and de los Campos, 2014). For all predictors except BGLR models, all features are combined into a single matrix of features for each sample. For BGLR models we provided markers, non-linear features, blocking features, and interactions as separate random effects, and covariates as fixed effects (i.e., using a uniform prior) to predict. For tree-based methods we did not consider pre-processing of scaling, non-linear or interaction features as these methods natively handle interactions and are unaffected by input range. For support vector machines we did not consider non-linear combinations or interactions of features as the SVM kernels handle this. K-nearest neighbour methods did not include the distance matrix features. For the BGLR mixed models, we also considered the NOIA additive and dominant encoding scheme (Ma et al., 2012) and epistasis similarity matrices are calculated as the Hadamard product of NOIA matrices (Vitezica et al., 2017), where the similarity matrices are specified separately in BGLR with RKHS priors. Hyperparameters and models were optimised using Optuna. All code is available at: https://github.com/darcyabjones/selectml.

## Results

**FIGURE 1:**
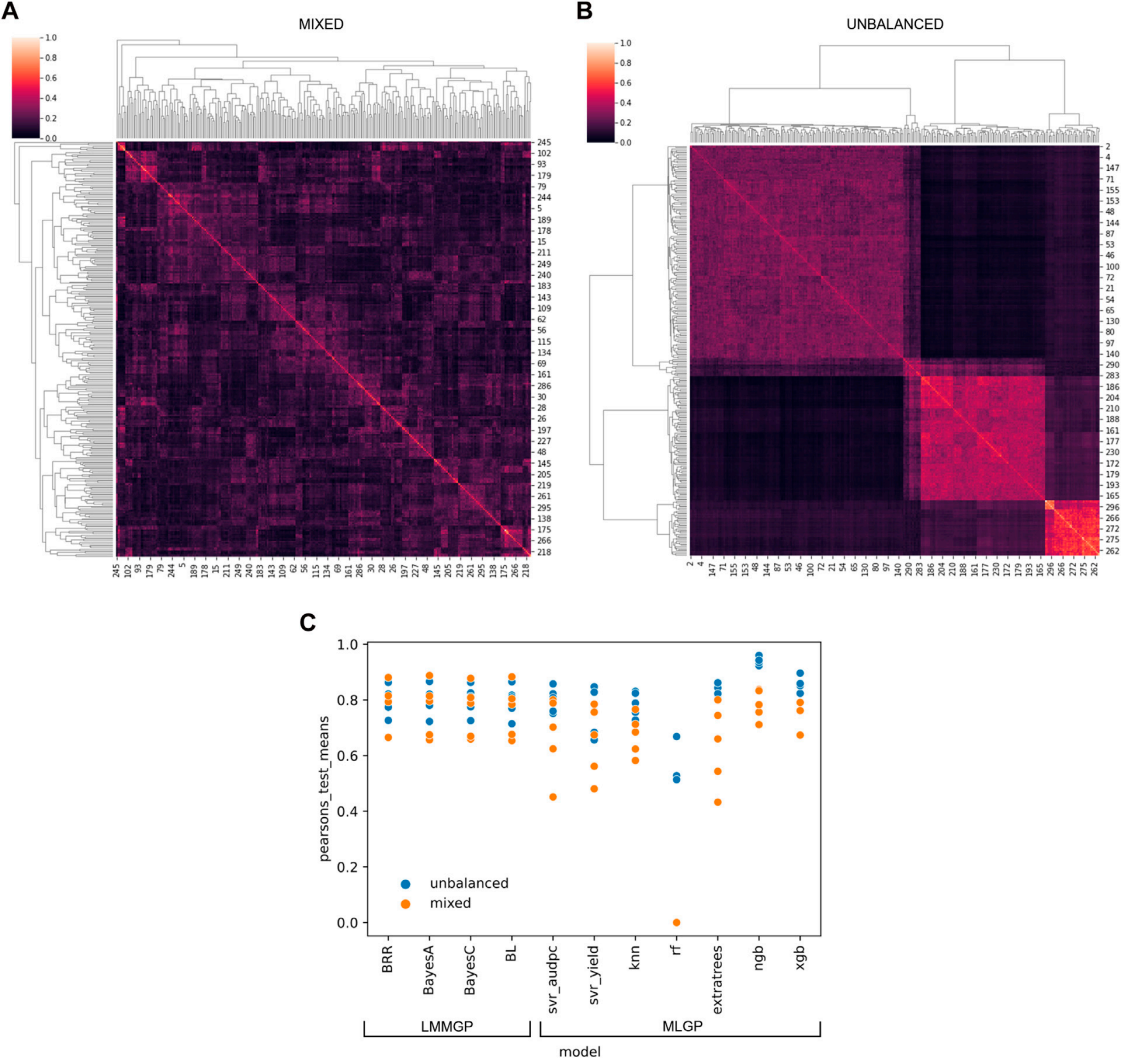
Application of multiple linear mixed model (LMM) and machine-learning (ML) genomic prediction (GP) tests via SelectML to two artificially-generated datasets simulating **(A)** a “mixed” dataset with random crosses from 40 parents, and **(B)** an “unbalanced” dataset with uneven sampling of 5 biparental populations. The results generated by SelectML **(C)** are presented in terms of the mean of Pearson’s tests for LMM-GP methods: Bayesian ridge regression (BRR), BayesA, BayesC, and Bayesian LASSO (BL); and ML-GP methods: Support Vector Regression (Svr_adupc, Svr_yield), k-nearest neighbors (Knn), random forest (RF), Extra trees, Natural Gradient Boosting (NGB), and eXtreme Gradient Boosting (XGB).

We developed software called “SelectML” (https://github.com/darcyabjones/selectml) to automate various steps in MLGP and LMMGP, including dataset reduction and model optimisation, and enabling their comparison (Jones et al., 2023). Relative performance, assessed via Pearson’s test for both simulated datasets, showed LMMGP methods performed consistently better overall than most of the MLGP methods included in SelectML (Figure 1). MLGP marginally outperformed LMMGP methods versus the unbalanced dataset, but conversely worse performance of MLGP versus the mixed dataset, suggesting MLGP was more sensitive to sampling biases represented by the two datasets. Of all MLGP methods tested, Natural Gradient Boosting performed best against both datasets.

## Discussion

There currently appears to be few advantages to using MLGP over LMMGP, with further improvements needed to deliver step-change improvements in crop genetic gain. Other studies have reported similar findings where relative performance of MLGP and LMMGP has been (disappointingly) similar, highly susceptible to dataset structure and experimental design, and generally non-transferrable to new datasets (Aono et al., 2022; Danilevicz et al., 2022; Gill et al., 2022; Jubair and Domaratzki, 2023). This initial lack of progress may not yet rule out the capabilities of MLGP with further development. However, specialised and multi-disciplinary expertise is needed to innovate new MLGP methods, and complex models may not yet benefit from MLGP until marker dataset size significantly increases or additional complementary data can be integrated. Pre-trainable NN models may offer opportunities to test integration of complementary data that LMMGP cannot handle (e.g., environment, images, time-series) or the integration of predicted genomic features derived from bioinformatics analyses. Pre-trained models may also enable integration of large external data sources potentially expanding the scope of a study to many more samples than would be available to a single breeding program. With an appropriate learning objective, pre-trained models can learn compressed generalised representations of input data that contain information relevant to the target task. This latent representation (typically the output of the layer before the final predictor layer in an NN) can then be used to initialise a model to learn the “real” target objective (known as finetuning). Leveraging this external data and latent representations would allow the use of more complex models with generalised information, while being less prone to overfitting with fewer samples. Larger datasets may also require complementary development of memory management methods, e.g., “mini-batching” or informed dataset reduction.

Recent development in attentional and graphical neural networks (e.g., transformers) used in natural language processing may offer new MLGP alternatives. Because loci and markers are represented as embeddings rather than fixed column positions, these new methods offer possibilities to integrate date from multiple genotyping experiments and potential to pre-train large “pan-genome-scale” models. Additionally, there is a conceptual similarity between restricted attention matrices and covariance structures used in LMM, and the inclusion of environmental blocking factors and covariates as embeddings can enable the model to share information between experiments. Generalisable and memory efficient neural network architectures, such as Perceiver (Jaegle et al., 2021), may allow broader applicability across datasets through pre-training and finetuning, but was not included in SelectML (Jones et al., 2023) due to its complexity and computational bottlenecks. We developed an GP implementation of Perceiver (https://github.com/darcyabjones/gperceiver) which in initial tests performed similarly to SelectML (Jones et al., 2023). However the potential to integrate complementary sources of data and pre-train models to predict numerous complementary tasks, followed by fine tuning the model to predict target phenotypes, is the primary novelty and benefit of this model. We have likely not yet tested its full potential for integrating rich external datasets during pre-training, e.g., genome-based predictions of functional annotations (Consortium, 2019; Paysan-Lafosse et al., 2023) or gene expression data. It may also allow for more variability in marker data, for example, a model could be trained on a set of markers and subsequent predictions made using a subset or new markers, and could trivially allow representations of polyploid data, multi-allelic markers, and complex markers (i.e., insertion-deletions).

The power of GP lies in the analysis of large genetic datasets *en masse* to predict phenotypic outcomes at a broad level. This does not require specific knowledge of the contributing genotypes and their biological functions and thus bypasses significant research bottlenecks. This underlying philosophy may be why examples of novel integrations of GP with genome-based bioinformatics are relatively rare. Genomics often employs an opposing philosophy of first determining whole genome sequences, followed by comparatively laborious prediction and/or experimentally validation of loci of interest. Perhaps this is why only recently examples can be found of hybrid methods that leverage the strengths of genomics to address inherent flaws affecting both MLGP and LMMGP. High-throughput genotyping methods (e.g., DArT-seq and SNP-chips) typically capture imperfect markers with LD-based phenotypic association, which are themselves a very small subset of markers in the dataset. Additionally, marker selection based on filter methods (e.g., ReliefF) or penalised models (e.g., LASSO) are both strongly affected by multicollinearity, while GWAS-based feature selection are often conservative and only select markers with an additive contribution. As imperfect markers become unlinked across generations and dissimilar populations, GP models do not generalise well and must be trained specifically for a particular dataset. Causal or perfect markers are highly valuable, being unaffected by feature selection or LD decay issues. The advantage of replacing conventional markers with whole-genome sequences, is that the latter should contain all perfect/causal markers. Despite this, recent attempts to integrate whole-genome data only slightly improved prediction accuracy (Ros-Freixedes et al., 2022), presumably as both genomic and marker datasets contain comparable levels of background noise. Alternatively, bioinformatics can either guide biologically-informed dataset reduction or assignment of biological priors before GP is performed. This may incorporate prediction of gene-based functional annotations [e.g., gene ontologies (GOs), conserved domains (Consortium, 2019; Paysan-Lafosse et al., 2023)]. Recent methods incorporating GOs as biological priors show promising improvements to prediction accuracy (Farooq et al., 2021). Future development of methods for routine integration of genome bioinformatics to enable pre-GP dataset reduction and feature selection may be able to capture a higher proportion of causal marker candidates from large genome-derived datasets, significantly improving the outcomes of both MLGP and LMMGP methods.

## Data Availability

The datasets presented in this study can be found in online repositories. The names of the repository/repositories and accession number(s) can be found below: https://figshare.com/articles/dataset/GRDC_-_CCDM_CIC_genomic_prediction_report/20069921?file=35902358.
